# The Efficacy of Yeast Supplementation on Monogastric Animal Performance—A Short Review

**DOI:** 10.3390/life13102037

**Published:** 2023-10-11

**Authors:** Vetriselvi Sampath, Shanmugam Sureshkumar, In Ho Kim

**Affiliations:** 1 Department of Animal Resource and Science, Dankook University, Cheonan 330-714, Republic of Korea; suve2314@gmail.com (V.S.); sureshbiogenetic@gmail.com (S.S.); 2 Smart Animal Bio Institute, Dankook University, Cheonan 330-714, Republic of Korea

**Keywords:** yeast, byproducts, pigs, poultry, performance

## Abstract

Due to a continual growth in the world’s population and the prohibition of antibiotics in animal production, the livestock industry faces significant challenges in the global demand for meat, eggs, and dairy products. The growing demand for organic products and the prohibition on antibiotic growth promoters (AGPs) have compelled animal nutrition experts to search for natural substitutes that include medical plants and beneficial microorganisms. Natural feed additives like probiotics are found to be more effective than AGPs in reducing the load of harmful intestinal pathogens. One of the probiotics that has generated considerable interest since ancient times is yeast. Yeast is used as a supplement in animal feeds due to its relatively high protein, amino acid, energy, and micronutrient content. Yeast byproducts such as yeast cells and cell walls contain nutraceutical compounds (i.e., β-glucans, mannooligosaccharides, and nucleotides) and have been shown to improve animal growth performance and health. Though the application of yeast supplements has been reviewed to date, only a scarce amount of information exists on the yeast-derived products in non-ruminant nutrition. Additionally, it is difficult for nutritionists to differentiate the characteristics, composition, and optimal feeding among the diverse number of yeast-containing products. Due to the increasing popularity of using yeast-based products in animal feeds, the development of analytical approaches to estimate yeast and its components in these products is greatly needed. Thus, in this review, we intend to provide current knowledge of different categories of commercially available yeast and yeast-derived additives, along with their role in improving animal growth performance and health, their proposed mechanisms of action, and the challenges of quantifying yeast content and biologically active components.

## 1. Introduction

Over the last few decades, poultry and pork meat consumption has steadily increased, and it is still expected to inflate in the future. Genetic selection has greatly improved production efficiency to meet market demand. However, it is recognized that high-growth and productive animals must possess optimal health to reach their full genetic potential [1]. Among the strategies adopted to maintain animal health, nutrition plays an important role. From this perspective, numerous feed additives and nutraceuticals have received considerable attention due to their potential [2]. The evaluation and adoption of management changes, including the use of antibiotic-substituted feed additives, is well established in livestock production systems worldwide [3]. Concerns about antibiotic residues in meat and the reduced susceptibility of bacteria to antibiotics (i.e., increased resistance to antibiotics by bacteria) inevitably lead to spending more money on reduced efficacy from less effective medications with the potential to impact human health.

The progressive reduction and, in many cases, the complete withdrawal of antibiotics have now transitioned to the increased use of an array of specialty feed ingredients and in-feed additives, along with appropriate vaccination programs plus other management changes [4]. The enhanced scientific credentials and use of antibiotic alternative products have helped to facilitate the adoption of the responsibility and reduction principles of “antimicrobial stewardship”. Though the food-producing animal industry has a huge range of antibiotic alternative products to assess and select for use in various production and management systems, the use of several “bioactive” antibiotic replacement products has taken the top spot, including prebiotics, probiotics, and organic acid additives that highly support intestinal epithelial integrity and modulate the immune system of animals [5]. One of the probiotics that has generated considerable interest in recent research is yeast. Yeast and its derived products were frequently used as feed additives in animal nutrition [6]. The primary use of these in feed additives in livestock diets is to provide essential nutrients, increase the palatability of the feed, improve their growth performance, and optimize the utilization of the feed [7]. With increasing industry standards and consumer awareness, as well as demand for healthy food products of animal origin, there is increased pressure on the industry for more natural and non-residual alternatives than conventional feed additives. Many researchers have demonstrated the beneficial effect of dietary probiotics in livestock diets [8]. Particularly, yeast serves as a natural enhancer of growth in the context of livestock nutrition and has undergone detailed research for many years. Various kinds of yeasts and yeast-based products are produced, marketed, and utilized in the formulation of animal feed [9]. Yet, considerable research has been conducted to evaluate the potential animal growth performance and health benefits of adding yeast, yeast derivatives, and yeast-containing ingredients, particularly when animals are housed in poor sanitation and/or suffer from a disease [6]. Therefore, this review focuses on the effects of different types of yeast products, their functional characteristics, and their application in monogastric animal nutrition. We also aim to offer guidance for the advancement and utilization of yeast-based feed products that can be advantageous to the livestock sector.

## 2. A General Description of Yeast

Yeasts are single-cell eukaryotic microorganisms that are classified in the kingdom of fungi [10]. They contain almost the same organelles as mature eukaryotic cells. Additionally, they are categorized as facultative anaerobes, indicating their ability to thrive and proliferate in both oxygen-rich and oxygen-depleted environments [11]. Microscopic fungi typically measure around 3–4 μm in dimensions, possess a nucleus encased in a membrane, and exhibit a variety of shapes, ranging from spherical to oval and filamentous. Such yeasts are found worldwide in soil and plant surfaces and are particularly abundant in sugary media such as flower nectar and fruit. Yeasts from the soil and from the skins of fruits and berries have been shown to dominate fungal succession during fruit decay and have resistance to antibiotics, sulfamides, and other anti-bacterial agents [12]. This resistance happens genetically and naturally and is not feasible to modify or transmit to other microbes. The main method of yeast reproduction is primarily budding, and occasionally fission, and these do not form spores in or on a fruiting body, while they can be identified and characterized based on cell morphology, physiology, immunology, and molecular biology techniques.

### Saccharomyces cerevisiae

Yeasts play a crucial role in producing substances with probiotic properties, including both live strains and modified forms of their cell walls. These formulations exhibit established immunosuppressive effects in livestock and also result in gastrointestinal functioning enhancements that ultimately contribute to better production outcomes [13]. Yeasts are naturally present in grains, grain co-products, silage, and hay fed to animals [14]. Previously, more than one thousand yeast species have been recognized, but very few have been exploited commercially, with *Saccharomyces cerevisiae* (*S. cerevisiae*) being predominant [15]. The majority of yeast species neither confer advantages nor disadvantages to humans and animals, while other specific species such as *S. cerevisiae*, *Kluyveromyces marxianus*, and *S. boulardii* offer positive effects [16,17], whereas *Candida*, *Cryptococcus*, *Torulopsis*, and *Trichosporon* [18] are considered as pathogenic. *S. cerevisiae* has been widely used in animal diets [19]. It is rich in polysaccharides, vitamins, proteins, amino acids, small peptides, nucleotides, and some growth factors. Pang et al. [9] demonstrate that yeast can improve the production performance of animals, thereby promoting their intestinal health, regulating intestinal flora balance, improving immunity, and improving meat quality traits. Notably, *S. cerevisiae* has an extensive history of application in the area of food processing. In particular, they have been known as baker’s yeast or brewer’s yeast for centuries as leavening for bread and as a fermenter of alcoholic beverages and wine production, respectively. Such yeast has been demonstrated as a natural feed additive in ruminant and non-ruminant animals for manipulating the gastrointestinal tract and the rumen environment. Previously, Chowdhury and Knabe [20] investigated the impact of adding *S. cerevisiae* yeast and aspartic acid to the diets of nursery pigs. The researchers examined growth performance as well as nutrient utilization in the pigs. They found that the inclusion of *S. cerevisiae* yeast in the diet led to improvements in growth performance and nutrient utilization, suggesting that the yeast had a positive effect on the overall health and development of the non-ruminant animals.

## 3. Yeast (*S. cerevisiae*): Mode of Action

**Figure 1:**
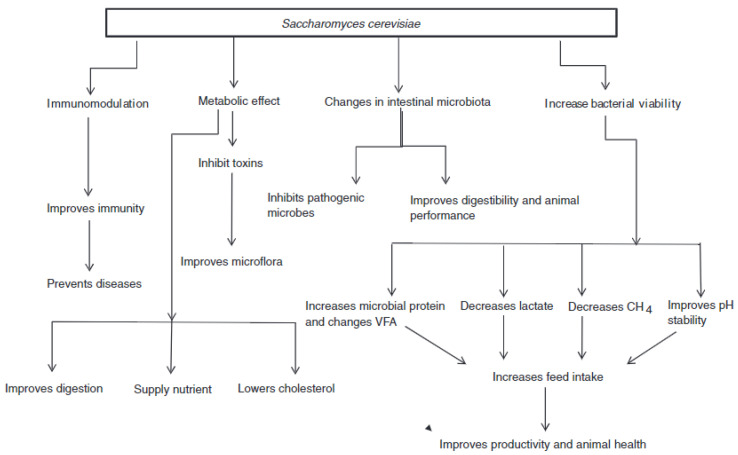
Overview on the mode of action of *Saccharomyces cerevisiae* as feed additives in livestock production. Source: Elghandour et al. [21].

Supplementation of yeast as a probiotic in livestock production uses various pathways (Figure 1) to employ their positive effects. In addition to the mechanisms and health benefits of yeast, they can be identified in animal production, which can be attributed to their ability to meet the criteria mentioned below [21]. The mode of action of *S. cerevisiae* (yeast) in diverse applications, such as non-ruminant feed, involves several beneficial interactions within the animal’s digestive and immune systems. Here is an overview of the key mechanisms:

**Competitive Exclusion**: *S. cerevisiae* competes with pathogenic bacteria for attachment sites on the intestinal lining. By binding to the yeast surface, harmful bacteria are prevented from adhering to the gut wall, reducing their ability to colonize and cause diseases.

**Improved Gut Health**: Yeast supplementation can lead to an enhanced gut environment by promoting the growth of beneficial bacteria while inhibiting the proliferation of harmful microorganisms. This rebalancing of the gut microbiota contributes to overall gut health and function [22].

**Stimulation of the Immune System**: *S. cerevisiae* contains cell wall components and compounds that stimulate the host’s immune system. These components activate various immune responses, including the production of immune cells and molecules that help with recognizing and combating potential pathogens [23].

**Enhanced Nutrient Utilization**: Yeast products contain enzymes such as phytase that aid in breaking down complex nutrients like phytate, which helps to improve the digestibility and absorption of essential nutrients such as minerals and amino acids [24].

**Production of Metabolites**: During fermentation, *S. cerevisiae* produces metabolites such as short-chain fatty acids, which positively influence gut health and contribute to the development of a favorable gut environment [25].

**Reduced Toxin Absorption**: Yeast can bind to certain toxins and mycotoxins present in feed, preventing their absorption in the gut and reducing their negative impact on animal health [26].

**Enhanced Intestinal Morphology**: Yeast supplementation has been associated with improvements in gut structure, including increased villus height and crypt depth. These changes can enhance nutrient absorption and overall gut integrity [27].

**Stress Alleviation**: Yeast products may help mitigate the negative effects of stress on animals. By supporting gut barrier function and immune responses, yeast can contribute to the animals’ ability to cope with various stressors [28].

In a study conducted by Buts et al. [29], it was observed that incorporating *S. cerevisiae* into the diets of monogastric animals led to the enhancement of their immune response. Majtan et al. [30] reported that dietary *S. cerevisiae* stimulates the synthesis and release of pro-inflammatory cytokines from macrophages. According to research by Qamar et al. [31], *Saccharomyces cerevisiae* was found to impact the immune response by promoting the production of IgA antibodies in response to pathogenic microorganisms. Certain strains of *S. cerevisiae* were noted to secrete a serine protease that can effectively break down toxin A derived from *Clostridium difficile*, as observed in the study by Castagliulo et al. [32]. Additionally, the presence of a substantial amount of methionine in *S. cerevisiae* enables it to mitigate the detrimental impacts of aflatoxins in animals, as indicated by Stanley et al. [33].

## 4. Benefits of Yeast Byproducts for Monogastric Animals

There are numerous forms of yeast and yeast derivatives available on the market, including viable yeasts, which are administered for their probiotic activity, and fractionated yeasts (i.e., yeast cell components, such as *β*-glucans and mannans), fed for their prebiotic activity [34]. Yeasts are available as specialty yeast products, such as selenium yeast and nutritional yeasts, that are particularly used for their high nutrient contents (proteins, amino acids, energy, and micronutrients) [6]. Though pre- and pro-biotic yeasts have been widely employed in the livestock sector, the research on yeast products such as yeast hydrolysate (YH) and yeast culture (YC) is still limited.

(i) **Yeast hydrolysate**: YH is a byproduct of hydrolysis extraction that naturally contains yeast extracts as well as yeast cell walls [35]. YH originates from *S. cerevisiae*, offering a cost-effective advantage when compared to other yeast extracts used as additives. It is rich in protein [11]. It can be obtained through different procedures. Autolyzed yeasts are extensively used by the livestock industry, particularly to increase feed palatability and support animals to achieve better health and digestion. Subsequently, hydrolyzed yeasts serve as an alternative protein source for animal feed and offer both nutritional and functional benefits to support the growth and health of young animals. Fue et al. [36] found that YH supplementation improved growth performance, serum immune cytokine levels, and increase the beneficial bacterial populations within the cecum of growing-finishing pigs. Furthermore, YH supplementation in the diet led to enhanced antioxidant capacity, disease resistance, and non-specific immunity in aquatic animals [37,38]. Wang et al. [39] found that adding 100 to 150 mg/kg YH to broilers resulted in improved intestinal morphology, barrier, and anti-inflammatory properties while decreasing intestinal permeability.
(ii) **Yeast culture**: YC is a kind of unique product. It is a combination of yeast biomass and fermentation metabolites formed during a specific fermentation process. Apart from that, it is created by inoculating live yeast cells on particular culture media and fermenting them under specific conditions, after which the entire fermented media is dried. The metabolic products may differ depending on the varied media and fermented conditions. It was reported that yeast cells ferment the sugars present in the culture media and the metabolic products include peptides, alcohols, and organic acids [6]. Previously, many studies reported that the inclusion of YC in swine and poultry revealed better performance. For example, Gao et al. [40] observed that adding *S. cerevisiae* YC to the diet resulted in enhanced growth performance, improved digestibility of calcium and phosphorus, and alterations in the intestinal mucosal structure in broiler chickens. Similarly, Shen et al. [41] demonstrated that incorporating *S. cerevisiae* YC in the diet improved growth performance, increased villus height, boosted the immune response in the gut, and enhanced nutrient digestibility in weaning pigs. However, some inconsistent results were observed in Wang et al. [42] and Yu. [43] studies, since the application of *S. cerevisiae* YC showed no improvements in poultry performance; thus, more studies are necessary to elucidate the underlying mechanism of its efficacy. Hooge et al. [44] and Stanley et al. [45] previously confirmed that YC has the potential to be an antibiotic-free choice in monogastric animal feed.
(iii) **Live yeast**: Due to their potential probiotic properties, live yeast products are generally added to animal feed as direct-fed microorganisms. Active dry yeast (ADY) is most widely used as a commercial yeast product in livestock diets because it contains 1.5 × 10^11^ live yeast cells/g CFU [46]. Tunnel dried yeast (granular powder), fluid-bed dried yeast (quick-rise yeast in oval-shaped spheroids), and roto-louver dried yeast (small spheres or balls) are the three processes utilized to obtain ADY [47]. Zhang et al. [48] previously reported that ADY has a positive role in the stomach, duodenum, small intestine, and cecum by reducing the growth of dangerous bacteria and boosting the propagation of beneficial bacteria. Due to the decreased damage in yeast cells, the fluid-bed drying method has become the most popular in the drying process. The European Food Safety Authority (EFSA) stated that *S. cerevisiae* yeast received more attention all over the world due to its health benefits. Particularly after the ban on certain antibiotics, *S. cerevisiae* yeast gained attention as an AGP in animal nutrition [49]. Live yeast, when serving as a probiotic, can operate through two distinct mechanisms: firstly, by exhibiting probiotic characteristics, and secondly, by providing beneficial protein, essential B-complex vitamins, and crucial trace minerals for the production of extracellular enzymes [50].
(iv) **Yeast extract**: Yeast extracts are widely used as in-feed additives or flavorings for the production of soup and meat products, which consist of YC content without the cell walls. Additionally, yeast extract finds applications in cosmetic ingredients, animal feed, and microbiological growth mediums [51]. There are two main methods used for YE production: autolysis and hydrolysis [52]. Autolysis is a conventional breakdown mechanism in which the yeast’s own cell enzymes are activated to disrupt cell components. The result of autolysis is yeast autolysate, which contains both intercellular and cell wall fractions [53]. Yeast autolysates (YA) are predominantly employed in the food industry to enhance flavors. However, research on the positive impacts of supplementing poultry diets with *S. cerevisiae* YA remains relatively scarce [54]. The most effective approach for solubilizing yeast involves hydrolysis, which can be achieved through the use of acids or enzymes [55].
(v) **Yeast cell wall**: Yeast cell walls (YCWs) can be classified into fractioned yeast products, whereas active components of *S. cerevisiae* yeast cell walls, such as mannan oligosaccharides (MOS) and beta-glucans, are often referred to as the best in-feed additives for animals [56]. The YCW components of *S. cerevisiae* represent about 15–20% of the dry weight, and almost 75% of cell walls are made up of polysaccharides. The cell wall contains three major polysaccharides: glucans, mannoproteins, and chitin [57]. Glucans (mainly beta-glucan) are the primary component of *S. cerevisiae* yeast cell walls, which are highly insoluble and represent 60% of the cell wall dry mass. It is well known that *S. cerevisiae*-derived beta-glucan could be an immunomodulator as it has strong effects on the animal immune system [58]. Mannans account for around 40% of cell dry mass and are regarded as the second most essential component of *S. cerevisiae* YCW [59]. MOS are derived from the outer layer of *S. cerevisiae* yeast cell walls and serve as prebiotics [60]. Previously, numerous researchers investigated the benefits of MOS dietary supplementation in farm animals and discovered that it had a beneficial influence on growth, health, and mortality [61]. These advantages may be ascribed to reduced intestinal pathogen counts, immune system modulation, improved intestinal mucosa integrity, and antioxidant, antimutagenic, and antigenotoxic protective actions [62].

## 5. Dietary Application of Yeast Supplementation in Swine and Poultry

**Figure 2:**
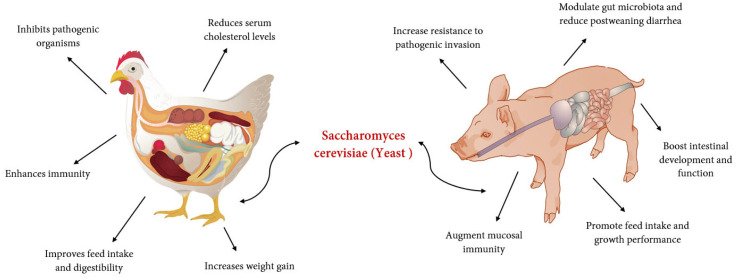
Beneficial effect of yeast (*S. cerevisiae*) supplementation on nutritional performance of monogastric animal (poultry and swine).

Yeast and its derivates have long been used in swine and poultry feeds. Table 1 shows the efficacy of yeast supplementation on health performance in swine and poultry. An earlier study demonstrates that the inclusion of 0.125% *S. cerevisiae* improved daily gain and gain-to-feed ratio (G: F) in weaning pigs [6]. Additionally, Price et al. [63] noted that 0.2% of dietary *S. cerevisiae* yeast (Diamond V XPC) increased the body weight gain of pigs challenged with *salmonella.* According to Shen et al. [41], the inclusion of 5 g/kg of yeast in the diet of weaning pigs led to a notable enhancement in the apparent total tract digestibility of dry matter, gross energy, and crude protein. Zhang et al. [64] reported that dietary yeast hydrolysate linearly improved daily gain, gain-to-feed ratio, and the digestibility of DM, GE, and nitrogen in finishing pigs. Though previous studies addressed the benefits of adding yeast to weaning pigs, the available data on the addition of yeast products i.e., YH, particularly in weaning and finishing pigs, is still limited; thus, further studies are needed to investigate the different types of *S. cerevisiae* yeast in grow-finishing pigs. *S. cerevisiae* yeast products not only improve the performance of pigs but also enhance the growth performance and nutrient digestibility of poultry. The beneficial effect of yeast (*S. cerevisiae*) supplementation on the nutritional performance of poultry and swine shown in Figure 2. For example, Bradley and Savage [65] reported that dietary inclusion of 10 g/kg of yeast cells increased gross energy and calcium and phosphorus retention in turkey. Similarly, Fath et al. [66] specified that the inclusion of yeast (comprised of glucomannan a natural aflatoxin-contaminated diet) increased feed intake, live weight, and feed conversion ratio in broilers. Nonetheless, findings by Sauer et al. [67] indicated that the addition of dietary *S. cerevisiae* yeast nucleotide has no impact on the digestibility of nutrients in the ileum of weaning pigs. Additionally, Yalcin et al. [68] reported that Hy-line brown laying hens fed a diet supplemented with *S. cerevisiae* yeast autolysate products did not show an improvement in body weight or feed efficacy. Gao et al. [40] found no differences in growth performance between broilers fed 0.25% and 0.5% SC-YC, and the 0.5% *S. cerevisiae* YC treatment had decreased ADG compared to the CON group from days 1 to 21. The effects of *S. cerevisiae* yeast products on swine and poultry growth performance and nutrient digestibility are sometimes contractionary. This may be due to the different product types, nutrient components, ages, and species of animals.

### 5.1. Effect of Yeast Supplementation on Gut Health

The gastrointestinal tract (GIT) of animals is a complex system consisting of numerous bacteria. It promotes nutritional digestion and absorption, prevents pathogen colonization, and maintains normal mucosal immunity, all of which contribute to host nutrition and health. Previous studies reported that yeast products have the ability to promote the intestinal microbiome in poultry and pigs. For example, White et al. [69] demonstrated that *S. cerevisiae* yeast-derived MOS increased the intestinal *lactobacillus* counts and decreased the *Bifidobacteria* count in weaning pigs. Moreover, Stanley et al. [45] reported that yeast reduced the population of intestinal *coliform* in broilers. Similarly, Zhang et al. [48] demonstrated that the yeast cell wall supplement improved the villi height, crypt depth, and villus height in broilers. Gao et al. [40] also noted that the inclusion of 2.5 g/kg of yeast supplementation increased VCR in the duodenum, jejunum, and ileum in broilers. On the other hand, Sauer et al. [67] stated that yeast nucleotides failed to influence the ileal digesta of piglets. Due to various effective ingredients and kinds of products, the mode of action of yeast in swine and poultry could not be standardized. However, it can be postulated that improvements in the gut microbiome and intestine morphology could be attributed to the binding and limitation of the colonization of pathogens in the GIT, which helps to improve the integrity of the intestinal mucosa and enhance immune system activity.

### 5.2. Effect of Yeast Supplementation on Fecal Gas Emission

Manure management is a major contributor to greenhouse gas emissions from pig farming, accounting for 18% of total greenhouse gas emissions from the livestock industry. Moreover, pig manure is associated with environmental pollution due to the presence of ammonium and hydrogen sulfide, both of which are considered harmful gases. The heightened concentration of NH_3_ and H_2_S poses a health hazard not only to animals but also to individuals working with them. Numerous research endeavors have been undertaken to investigate dietary interventions aimed at mitigating environmental risks arising from the release of noxious gases like NH_3_, H_2_S, and methyl mercaptan [70]. For example, Sampath et al. [71] reported that in piglets fed a diet supplemented with a mixture of blood plasma and yeast, NH_3_ and H_2_S emissions were significantly reduced. Likewise, Li and Kim [72] noted a reduction in hydrogen sulfide (H_2_S) emissions among growing pigs when their diet was supplemented with a cell-wall product derived from *S. cerevisiae*. The study by Yan and Kim [73] stated that there was a strong connection between fecal noxious gas emissions and nutrient digestibility. The fecal gas emission content is often associated with nutrient digestibility and enhanced digestion, which may lower substrates of microbial fermentation in the large intestine and result in lower gas emission content [64].

### 5.3. Effect of Yeast Supplementation on Meat Quality

The compositional quality (lean-to-fat ratio) and palatability aspects such as visual appearance, firmness, juiciness, tenderness, and flavor describe the quality of meat. The quality of pork can be determined by a combination of genetic and environmental factors. While slaughter weight greatly affects the production cost, the major criteria to measure the quality of pork meat are marbling, color, pH, and water-holding capacity. Along with this, backfat thickness has a reflection on lean meat percentage (LMP) and assists in evaluating the meat quality. Determining the meat quality is always a complex concept, as it depends on consumer preferences. According to Ismail et al. [74], in order to generate optimal meat quality, the carcass must have a maximum meat yield with a minimum fat content. Certain findings indicated that including 0.3% yeast in the diet resulted in an enhancement of the antioxidant status in muscle tissue. Simultaneously, Li et al. [75] demonstrated that elevating the yeast levels in the diet led to reductions in both drip loss and the concentration of thiobarbituric acid-reactive substances in both muscle and meat. Numerous experiments have substantiated that the inclusion of yeast as a dietary supplement can lead to beneficial enhancements in the quality of broiler chicken meat. For instance, studies conducted by Bonomi et al. [76] and Lee et al. [77] demonstrated that broiler chicks fed diets enriched with yeast, specifically *S. cerevisiae*, showed improvements in meat tenderness and increased water-holding capacity. Previously, Maltin et al. [78] reported that meat color and tenderness are very important factors in determining quality because consumers often connect the color of meat with freshness, and the variations in tenderness may affect the decision of customers to repurchase. This statement was proved by Sampath et al. [71], who showed that in broilers fed YH supplements, cholesterol content was significantly reduced. Chicken meat is regarded as a readily available source of high-quality protein and other nutrients required for optimum body function. It is rich in calcium, magnesium, phosphorus, and sodium compared to pork meat. Previously, Lee et al. [77] noted that a chick-fed diet supplement with yeast enhanced the water-holding capacity and tenderness and reduced drip loss at day 7.

**Table 1:** Efficacy of yeast supplementation on health performance in swine and poultry.

Items	Level	Animals	Effects	Reference
Brewer’s yeast hydrolysate	0.1%, 0.5%, 1.0%, and 3.0%	Laying hens	Improved the egg production, egg quality, and nutrient digestibility of DM and N; enhanced the fecal microbiota of fecal *lactobacillus*; and reduced *E. coli* counts.	Park et al. [79]
Mixed yeast culture derived from *S. cerevisiae* and *K. maxianus*	0.1% and 0.2% mixed yeast culture (MYC)	Broiler	Supplementation of MYC improved the growth performance, enhanced the apparent total tract digestibility of DM, increased the contents of WBC and *Lactobacillus*, and positively influenced bursa weight in broiler chickens.	Sun and Kim [80]
Yeast extract complex (*K. maxianus* and *S. cerevisiae*)	0.1% and 0.2% yeast extract complex (YEC)	Weaning Pigs	Improved ADG and ATTD of DM in weaning pigs.	Shi and Kim [81]
Hydrolyzed yeast	0.5, 1.0, and 1.5 g/kg	Weaning Pigs	Improved BW, immunoglobulin secretion, and antioxidant enzyme activity, whereas it lowered diarrhea occurrence, lipid peroxidation, and pathogenic bacteria in weaning pigs.	Boontiam, et al. [35]
Hydrolyzed yeast	0.1% and 0.2%	Broiler	Significantly enhanced the growth performance of BWG and nutrient digestibility of DM and N; shifted Microbiota by raising excreta *Lactobacillus* counts; and decreased *E. coli* counts.	Sampath et al. [71]
Yeast hydrolysate (*S. cerevisiae*)	0.05% and 0.1%	Finishing Pigs	Increased growth performance, apparent total tract digestibility, *Lactobacillus* bacterial counts, BFT, and LMP in finishing pigs.	Sampath et al. [82]
Yeast autolysate (YA)	2 g/kg	Laying Hens	*S. cerevisiae* yeast autolysate product did not show an improvement in body weight and feed efficacy.	Yalcin et al. [68]
Yeast hydrolysate	50, 100, 150 mg/kg YH	Broilers	Dietary YH supplementation improved intestinal morphology, barrier, and anti-inflammatory functions, while it decreased intestinal permeability of broilers.	Wang et al. [39]
Yeast culture	2.5 g/kg, 5 g/kg, 10 g/kg, and 20 g/kg	Nursery pigs	YC improved growth performance of pigs, probably by improving villus height, gut immune response, and nutrient digestibility.	Shen et al. [41]
Yeast autolysate	1, 2, 3 and 4 g kg^−1^	Laying hens	Beneficial effects on performance, egg cholesterol content, and humoral immune response.	Yalçın, et al. [54]
Yeast (*S. cerevisiae*)	0.5% whole yeast (WY from SC), 0.3% SC extract (YE), and 0.3% SC cell wall (CW)	Broiler Chicks	SC cell components to broiler chicks could improve growth performance, meat tenderness, and oxidative stability of meat. It is the yeast cell wall, not the yeast extract, that could improve ileal mucosal development of broiler chicks.	Zhang et al. [57]
Brewer’s yeast hydrolysate	0.05%, 0.1%, 0.5%, and 1.0%	Growing-finishing pigs	Improved the growth performance with body weight and feed efficiency, besides the apparent digestibility of nutrients.	Zhang et al. [64]

## 6. Conclusions

In summary, numerous yeast products are produced and used in animal diets as nutritious sources. Previously published research findings have indicated that the utilization of different yeast products has the potential to enhance growth performance, nutrient digestibility, gut microbiome balance, intestinal structure, overall health, and production and reproductive attributes in swine and poultry. The positive effects of yeast products may be attributed to their potential to prevent the establishment of pathogens in the gastrointestinal tract, adjust the composition of the gut microbiota, influence the immune system, and potentially engage in antioxidant and antimutagenic activities. The effectiveness of yeast may vary due to factors such as the specific types of yeast used, dosage levels, nutritional qualities of the feed, environmental conditions, and the age and species of the animals involved. Hence, there is a requirement for further insights into the potential mechanisms of various yeast products when incorporated into swine and poultry diets. It is crucial for producers and consumers to have a comprehensive understanding of the inherent distinctions among these products, including their possible contributions when integrated into the livestock diet.

## Data Availability

The data presented in this study are available on request from the corresponding author.
